# Watt’s Chemical Essays

**Published:** 1867-08

**Authors:** 


### WATT'S CHEMICAL ESSAYS.

We are gratified to learn that Dr. S. S. White is about publishing, in book form, a collection of the various chemical papers of Prof. Watt, which have appeared from time to time in the journals, they being carefully revised by the author. Many of the profession have long felt the want of something of the kind. Those who have read these essays, as they appeared, will want to read them again; those who have not will now be able to obtain them in a desirable and permanent form. We believe Dr. White expects to have the work out in time for the fall trade. We will notice more fully, when better posted as to the facts.

